# RNA-Binding Proteins: Splicing Factors and Disease

**DOI:** 10.3390/biom5020893

**Published:** 2015-05-13

**Authors:** Alger M. Fredericks, Kamil J. Cygan, Brian A. Brown, William G. Fairbrother

**Affiliations:** 1 Department of Molecular Biology, Cell Biology, and Biochemistry, Brown University, 70 Ship Street, Providence, RI 02903, USA; E-Mails: alger_fredericks@brown.edu (A.M.F.); kamil_cygan@brown.edu (K.J.C.); b.anthony.brown830@gmail.com (B.A.B.); 2 Center for Computational Molecular Biology, Brown University, 115 Waterman Street, Providence, RI 02912, USA

**Keywords:** RNA-binding proteins, motif, splicing

## Abstract

Pre-mRNA splicing is mediated by interactions of the Core Spliceosome and an array of accessory RNA binding proteins with *cis*-sequence elements. Splicing is a major regulatory component in higher eukaryotes. Disruptions in splicing are a major contributor to human disease. One in three hereditary disease alleles are believed to cause aberrant splicing. Hereditary disease alleles can alter splicing by disrupting a splicing element, creating a toxic RNA, or affecting splicing factors. One of the challenges of medical genetics is identifying causal variants from the thousands of possibilities discovered in a clinical sequencing experiment. Here we review the basic biochemistry of splicing, the mechanisms of splicing mutations, the methods for identifying splicing mutants, and the potential of therapeutic interventions.

## 1. Introduction

Most genes in higher eukaryotes are composed of introns (non-coding segments) and exons (coding segments). The majority of human intron removals are catalyzed by a large and dynamic ribonucleoprotein (RNP) complex called the spliceosome. This process involves two sequential transesterification reactions which ligate the exons and release the intron as a lariat.

Alternative splicing allows for the production of many proteins from a single genomic locus. This greatly augments the repertoire of proteins that can be produced by a given gene and plays an important role in evolution, development, and disease. It is estimated that 95% of human genes are alternatively spliced [1]. The regulation of splice site usage is not well understood, but has been shown to be specific to species, populations within a species, and tissues within individuals [1,2,3]. Misregulation of splicing often results in exon skipping (truncation of the translated protein), intron retention (translation of non coding regions), or alternative splice site usage (alteration of protein composition). Changes in transcript levels or transcript ratios are generally more deleterious than substitutions and have been implicated in several RNA-dependent diseases [4,5,6].

Splicing is a sequential process facilitated by the interaction of *cis*-sequence elements and *trans*-acting RNA-binding proteins (RBPs). Splicing can be highly variable as mRNA-RBP interactions are transient and of relatively low specificity. Changes in *cis*-sequence and levels of *trans*-factors can alter splicing and cause disease. In fact, approximately one third of all disease alleles are thought to affect splicing [7].

### 1.1. The Core Spliceosome

**Figure 1:**
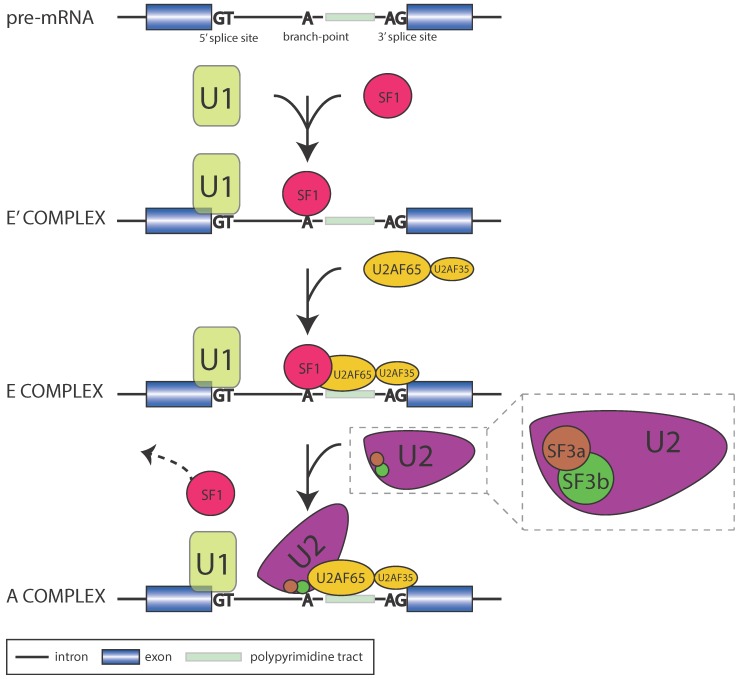
Stepwise assembly of the early splicesome highlighting the known splicing factors that bind to the substrate.

The major spliceosome is made up of five small nuclear ribonucleoproteins (snRNPs): U1, U2, U4, U5, and U6 and catalyzes ≈99% of splicing in humans [8,9,10,11]. The spliceosome is one of the more complex macromolecules in eukaryotic cells consisting of over 300 different proteins [12,13]. The 5' and 3' splice sites (ss) are recognized in a coordinated manner. Exon definition is initiated by the U1 snRNP which binds to the 5'ss motif, and the splicing factor SF1 which binds to the branch-point sequence just upstream of the 3'ss. This committs the pre-mRNA transcript to the splicing pathway and forms the commitment (E') complex [14]. Next an additional splicing factor, U2AF65, cooperatively binds with SF1 to recognize the polypyrimidine tract between the branch-point and 3'ss and the 3'ss itself forming the early (E) complex [15]. The U2 snRNP then displaces SF1 by base pairing at the branch-point motif in an ATP-dependent manner forming the ATP-dependent (A) complex (Figure 1). This process is catalyzed by the RNA helicases Prp5 and Sub2. The Prp5 helicase helps the base pairing interaction by binding the U2 subunit and stabilizing the branch-point-interacting stem-loop (BST) which actually base pairs with the intron [16,17]. Sub2 is necessary to stabilize the interaction between the RNA branch-point and the U2 subunit. U4, U5 and U6 tri-snRNP is then recruited to form the pre-catalytic spliceosome or the B complex [18]. This process is catalyzed by the DEAD-box helicase Prp28. This protein releases the U1 snRNP during the recruitment of the U4, U5,and U6 snRNP [19]. The pre-catalytic spliceosome goes through a series of conformational changes catalyzed by RNA helicases Brr2, Snu114, and Prp2, which lead to the release of the U1 and U4 subunits forming the activated B (B*) spliceosome. Brr2 helps retain U5 and U6 while releasing U1, Snu114 and Brr2 assemble on the U5 snRNA to produce the U5 snRNP, and Prp2 is responsible for destabilizing the RNA core of the spliceosome to catalyze the conformational change from the B complex to the C1 complex [20,21,22]. The subsequent splicing events take place in two steps. First, U2 associated protein complexes SF3a and SF3b are released and expose the branch-point allowing a nucleophilic attack by the branch-point 2'OH group on the 5' splice site. This results in a free 5' exon and a lariat intron intermediate (C1 complex) [23]. The second step of splicing is promoted by the Prp8 protein which cross links the U5 and U6 snRNP [24]. In this step 3' OH of the 5' exon attacks the 3'ss forming the C2 complex. The remaining snRNPs and associated factors are disassembled, the exons are ligated, and the intron lariat is released and rapidly degraded by the cell [25]. Exons can be constitutive (included in all isoforms of the transcript) or alternative (included in only some isoforms of the transcript), and the availability and recruitment of the associated splicing factors have been demonstrated to regulate this through influencing splice site efficiency (relative strength), and as a result splice site usage. The two major splicing factor RBPs are the Heterogeneous ribonucleoprotein particles (hnRNPs) and serine-arginine (SR) proteins. These two RBPs have opposite enhancing and repressive qualities that often depend upon where they bind.

### 1.2. hnRNPs, SR Proteins, and Other Splicing Factors

hnRNP proteins are a well characterized class of RBPs which perform their functions in large homopolymer complexes as opposed to diverse ribonucleoprotein complexes [26]. These aggregates are made up of major hnRNP proteins that form the core of the hnRNP aggregates, and minor proteins that are more transiently associated with a subset of hnRNP homopolymer complexes [27,28,29]. Although there remains a class of uncharacterized hnRNP proteins, the majority (over 50%) have been characterized to play a role in splicing [30]. Other functions include mRNA export, localization, translation, and stability. hnRNPs bound to exonic motifs function as splicing suppressors. hnRNPA1 for example binds to a high affinity RBP motif in the third exon of the HIV-1 gene. Additional hnRNPs are recruited, and the subsequent homopolymer inhibits splicing by disrupting spliceosome assembly [31]. hnRNP binding motifs in introns conversely have been shown to enhance splicing. hnRNPH has been shown to enhance splicing in the mouse *src* gene resulting in a neuron specific isoform [32].

SR Proteins are a large family of RBPs that were first described in the early 1990s by the Gall and Roth laboratories independently. The Gall laboratory identified four SR proteins (SRp20, SRp40, SRp55 and SRp75) [33] using the monoclonal antibody mAb104 against the phosphorylated epitope of the SR protein in *Xenopus laevis* [34]. Concurrently the observation of B52 antibody bracketed RNA polymerase II (Pol II) on Hsp70 loci of polytene chromosomes in *Drosphila melanogaster* provided a link between the B52 splicing factor and SF2/ASF, which was previously implicated in constitutive and alternative splicing [35,36,37]. Ultimately three SR proteins were identified: suppressor-of-white-apricot (SWAP) [38], Transformer (Tra) [39], and Transformer-2 (Tra-2) [40,41]. SR proteins are named for their conserved Arg/Ser (RS) binding domain, which distinguishes them from most other RBPs. This domain is found near the C-terminal domain of the protein and promotes protein-protein interactions between the SR protein and the spliceosome [42]. SR proteins have been shown to recruit and stablize interactions between: U1 snRNP and the 5'ss by bridging the U1-70K binding domain to the pre-mRNA transcript, U2AF and the 3'ss through U2 snRNP interactions, and U4/U6.U5 tri-snRNP and the pre-spliceosome complex by promoting the formation of the cross-intron complex [43,44,45]. Improper phosphorylation of SR proteins however has been shown to block U2 from binding the 3'ss and function as a splicing inhibitor [46]. Although hnRNPs and SR proteins are thought to be the major RBP regulating splicing associated factors, recently other RBPs have been implicated in influencing splicing.

RBPs from several other protein families with previously undefined roles in splicing have recently garnered great interest and are now being implicated as key splicing regulators. One example is the splicing factor FUS. FUS is a member of the FET protein family along with EWSR1 and TAF15 [47]. The function of FET family proteins have not been well characterized, but recent studies suggest that FUS is involved in transcription, splicing and mRNA transport, microRNA processing, DNA repair, and cell proliferation [48]. The C-terminal of FUS contains an RNA binding domain with several RNA binding motifs including three arginine-glycine-glycine boxes, a zinc finger, and an RNA recognition motif, though the exact residues involved in interactions with RNA are yet to be described in the literature. The N-terminus contains SYGQ rich domain which binds transcription factors and activates transcription through interactions with Pol II. As approximately 80% of splicing occurs cotranscriptionally [49], the interplay between Pol II activation and RNA binding makes FUS an interesting splicing factor candidate.

## 2. Three Mechanisms of RBP-Related Splicing Dysregulation

Here we describe three basic disease mechanisms caused by dysfunctional mRNA-RBP interactions: the disruption of *cis*-elements, toxicity conferred by mutant mRNA transcripts, and the loss of *trans*-acting factors.

### 2.1. Mechanism I: Disruption of a Splicing Element

Non coding point mutations that cause splicing defects constitute about 13.5% of heriditary disease alleles reported in the Human Gene Mutation Database (HGMD). A wide range of common human disease such as: Ataxia Telangiectasia, Retinitis Pigmentosa, breast cancer, and Cohen's Syndrome are caused by changes in splice site recognition [50,51,52].

The highly conserved GU/AG motifs mark the beginning and end of 99% of introns. Mutating either motif prevents the interactions between the core spliceosome and the pre-mRNA transcript that occur during the splicing process [9]. Most intronic point mutations annotated as splicing mutations fall within two nucleotides of the exon. Beyond the dinucleotide motif the core *cis*-splicing elements extend from the –3 position to the +6 position at the 5' splice site, and from the –20 position to the +3 position at the 3' splice site traversing the exon intron junctions. The remainder of the *cis*-sequence is significantly divergent with the probability of a base in any position ranging between 35%–80%. Less than 5% of splice sites match the consensus motif perfectly [53]. This poses the fundamental question of how exons are recognized in large introns. An additional degree of definition could come from a branch-point sequence which is required for splicing. Mutations in the branch-point sequence just upstream of the 3' splice site have been shown to have a similar effect in some heritable disorders [54,55,56]; however, the relatively low number of branch-point sequences that have been identified and the relative degeneracy of the motif in humans restricts the ability to screen for this class of variants in a high throughput manner [57]. Auxiliary elements could explain how splice sites are distinguished from the multitude of psuedo splice sites found in introns [58]. In the next section we describe how the disruption of auxiliary splicing elements contributes to deleterious variability in splice site usage.

**Figure 2:**
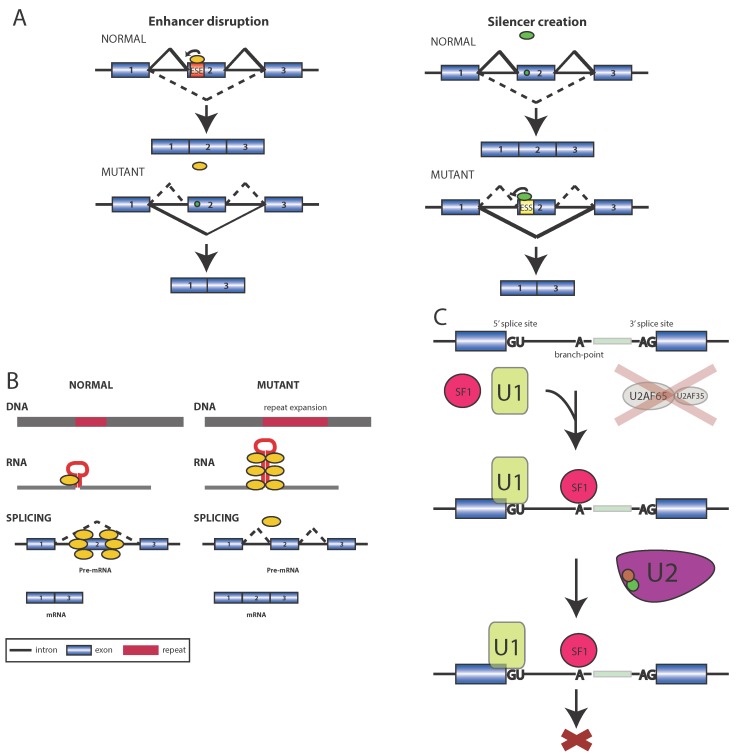
Three mechanisms of RBP-caused splicing dysregulation. (**A**) Disruption and/or creation of cis-elements by disease variants. (**B**) The RNA becomes toxic as a result of repeat expansion. Misregulation of splicing by the toxic RNA occurs through sponge-like titration of a splicing factor. (**C**) Mutation in splicing factor (e.g., U2AF) prevents it from binding to the pre-mRNA and stabilizing U2 snRNP. This results in unsuccessful transcript recognition.

Disease mutations can also alter splicing by the disruption of *cis*-elements that modulate the recognition of splice sites. These auxiliary elements are often ligands for RBPs. The principle splicing factors that bind these auxiliary enhancers and silencers are the SR and hnRNP protein families. Both protein families are generalized to function in a position specific manner. In other words, SR proteins bound in the exon are generally regarded as activating splicing whereas the same protein relocated to the intron can act as a repressor. Conversely, hnRNPs are regarded as repressors when bound to exonic locations and activators when bound to the intron. The binding specificities of many RBPs have been modeled *in vitro* and can be used to evaluate the potential of a variant to disrupt a binding site [59]. This position dependence seems to be a general property of splicing elements. Exonic splicing enhancer (ESEs) motifs functionally repress splicing when found in the intron, becoming intronic splicing silencers (ISSs) [60]. Likewise exonic splicing silencer (ESSs) motifs have been shown to function as intronic splicing enhancers (ISEs) (Figure 2A) [61]. Positional distribution analysis uses this property to predict loss of binding without knowledge of the *trans*-acting factor ([7], see also Spliceman below). Non-coding, and functionally conservative or silent mutations that have little to no effect on the translated protein have been demonstrated to cause disease by disrupting splicing [62]. In a recent mutational survey of HGMD, it was estimated that 25% of reported missense and nonsense mutations disrupt splicing by creating or destroying auxiliary exonic signals [63]. It is worth noting that causal alleles with mutations in auxiliary *cis*-sequence that disrupt splicing have also been identified in each disease previously described.

### 2.2. Mechanisms II: Toxic RNA

Mutations that increase the stability of interactions between an RNA species and RBP substrate can cause disease. This has been demonstrated in several well studied diseases particularly neurological and muscular degenerative disorders. The common feature that defines this class of disorders is repeat expansions that are particularly unstable and often result in further enlargement. Often, the repeated sequence becomes pathogenic after expanding beyond a threshold length. The toxic mRNA transcripts produced cause the dysregulation of alternative splicing of many pre-mRNAs in *trans* simultaneously. Also known as spliceopathy, this pathogenic mechanism has been observed in several RNA-dominant diseases including Myotonic Dystrophy (DM). Spliceopathy is observed when repeating motifs specify an RBP ligand. The repeat expansion creates a tandem array of RBP binding sites which recruits and sequesters RBPs to the transcript, resulting in a sponge like titration of splicing factors effectively depleting the available pool in the cell (Figure 2B). In an opposite fashion the expansion can also lead to the upregulation of RBPs that bind only to the short, endogenous motifs. An increase in the splicing factor CUGBP1, a CELF family protein specific to striated muscle, is also pathogenic in DM. CUGBP1 when unbound to its substrate becomes hyper phosphorylated, giving it a negative gain of function that contributes to extensive splicing dysfunction [64]. In DM, two distinct repeat expansions have been reported as the mechanism of pathogenesis: CUG in DM1 and CCUG in DM2 in non coding regions of the DMPK and ZNF9 genes respectively [4]. In both cases expression of the transcript is repressed, but the more significant pathogenic result is generated by the sequestration of many RBPs involved in mRNA biogenesis including splicing factors. Although patients share core phenotypes, DM in addition to many other degenerative disorders such as Alzheimer's and Spinocerebellar Ataxia present in a markedly variable composite phenotype. This may be explained by the broad yet relatively non specific impact of toxic mRNAs.

### 2.3. Mechanism III: Mutations that Affect Splicing Factors

The other major category of spliceopathy is direct mutation of a splicing factor (Figure 2C). Mutations in splicing factors have been described in a wide array of common diseases. A pair of the more well understood spliceopathic RBPs are NOVA (Paraneoplastic neurological disorders), and TDP-43 (ALS). Both splicing factors regulate alternative events in neurons, and the loss of either result in severe pathogenesis. NOVA belongs to the K-homology (KH) family of RBPs and is known to interact with hnRNPE1 and hnRNPE2 to promote inclusion of an alternatively spliced transcript [65]. The spliceopathy of TDP-43 however is conferred by its ability to regulate itself. TDP-43 governs splicing patterns of ≈950 transcripts, increase or decrease in cellular TDP-43 causes exclusion events in its target transcripts. The global loss of splicing regulation in the neuron is thought to result in aggregates of ubiquitinated inclusions [66]. In Dilated Cardiomyopathy (DCM) the RBP RBM20 has been shown to regulate diastolic function, sarcomere assembly, and ion transport in an enhancer dependant mechanism. RBM20 is recruited by phosphorylated SR proteins causing inclusion of differentially expressed (mutually exclusive) exons, which promotes elasticity primarily in the sacromeric titin protein. Depletion of RBM20 reduces cardiac elasticity causing heart disease [67]. RBFOX1 is a neuron specific splicing factor that is associated with with several neurodegenerative disorders including Autism. RBFOX1 plays an important role as a master regulator of splicing in the development of early neurons. Loss of the RBFOX1 causes changes in synaptic transmission as well as membrane excitability. Variants that deplete RBFOX1 show a globally negative affect on growth and proliferation in most neurodevelopmental pathways [68]. Finally, splicing factor RBPs such as SRSF1 have been strongly correlated with proto-oncogenic transformations. SRSF1 has been shown to regulate the splicing of several oncogenes. SRSF1's primary target, BIN1, is known to inhibit cMyc. Depletion of SRSF1 leads to an aberrant BIN1 protein with reduced ability to suppress cMyc [69,70]. Each of these RBP splicing factors perform different functions in splicing regulation and disease. This demonstrates the multitude of biological processes dependent on the regulation of pre-mRNA splicing.

## 3. Developing Tools Predicting Causal SNPs

Several tools have been developed to predict the effects of variants on splicing. These tools evaluate splice site strength (MaxEntScan) [71], predict splice site usage (NetGene2) [72], identify splice site motifs (RESCUE-ESE) [73], as well as predict the effect of mutations in both canonical splicing motifs (ASSEDA) [74] and auxiliary motifs (Spliceman) [75]. Spliceman for example, uses the positional distribution of hexamer motifs around exon intron junctions to predict variants outside of canonical splice site signals that disrupt splicing. However, the complex haplotype architecture of genetic variation in humans makes it challenging to functionally assess individual variants in the laboratory. The haplotype identified in an association study requires further analysis to find causal variants.

The cost and time of sequencing and analysis has dramatically decreased since the original genome-wide association studies (GWAS). Data is now produced in tremendous volumes and consolidated in databases. One such database, the database of Genotypes and Phenotypes (dbGAP) hosted by the National Center for Biotechnology Information (NCBI) combines genotype and phenotype data from the literature and the clinic. Some of this data like the Genotype Tissue Expression Project (GTEx) [76] combines a survey of RNA-seq data from different tissues with genomic sequencing data in a diverse population of individuals. Here, variants in individuals that are discovered within the cohort can be checked for changes in the individuals' transcript level or splice isoform usage. Although correlations between variants and processing defects do not necessarily prove causality, this type of data greatly reduces the search space for common variants that affect splicing. Furthermore planned expansions of this dataset: increasing the population size and diversity, and adding the dimension of RNA deep sequencing data should reduce false positives and allow for higher confidence in observed associations. Analysis of the GTEx data upon the completion of the project will likely lead to refined predictive tools and an increase in the identification of causal variants.

## 4. Functionally Validating Individual Variants

Despite the great promise of public datasets such as GTEx and predictive tools, experimental approaches offer the most definitive test of causality. For common variants, causality can be determined by testing variants in linkage disequilibrium (LD) with the associated single nucleotide polymorphism (SNP) in a splicing assay. This approach allows the effect of the variant to be measured independently of neighboring SNPs and to control for the genetic background. Here, we will discuss high throughput strategies we are developing in our lab to functionally evaluate variants of interest and attribute causality. These approaches can be applied to SNPs, disease alleles or variants of unknown significance that are returned in exome sequencing studies.

Minigene reporter constructs can be synthesized and used to identify variants in the *cis*-sequence that demonstrate allele-specific splicing defects [77]. Variation of the minigene constructs can be developed with alternate promoters and vectors, and be tested in a number of cell lines with different expression profiles to account for tissue-specific expression level and neighboring environment variability. Original minigene constructs relied on the generation and insertion of recombinant complementary DNA (cDNA) into the genome, but more recently simple polymerase chain reaction (PCR) strategies have been used to amplify sequence of interest from genomic DNA which are ligated to splicing reporters to measure splicing activity [78]. The main limitation to constructing minigene reporters from genomic DNA is the inability to separate nearby variants.

Currently, DNA libraries of short oligos can be synthesized to test the splicing efficiency of variants and their wild type pairs in a neutral background sequence. These minigenes can undergo splicing in nuclear extract or be transfected into cells to assay *in vitro* or *in vivo* splicing activity respectively [77]. Results can be directly quantitated by comparing the levels of input RNA and spliced product [79]. These mutant wild type pairs can then be tested for splicing activity in a massively parallel high throughput assay [79]. This approach is limited by the length of oligonucleotide that can be accurately synthesized by the current technology. We currently employ a combination of these strategies. Oligo libraries are designed to uncover candidate variants that influence splicing in a high throughput manner. We then validate the candidate variants by assessing splicing phenotype in patient-derived tissues by RT-PCR assay. A key advantage of high throughput functional assays is that their input can accommodate the typical number of variants called in an exome sequencing run (*i.e.*, 20–30,000).

Loss of *trans*-acting splicing factors and toxicity of RNA can be measured using well characterized binding assays such as immunoprecipitations, fluorescent *in situ* hybridization, and chromatography. In binding assays *in vitro* techniques allow for the direct comparison of intrinsic binding between different RBPs and their substrates in titrated concentrations and environments. Association kinetics in endogenous conditions likely vary significantly from *in vitro* assays. Conditions *in vivo* are the result of complex interactions between a multitude of factors and binding assays with rare or low concentrations of spliceopathic transcripts may not produce a discernable signal. Binding assays as well have been adapted to high throughput platforms to increase sensitivity and the amount of data produced. Low throughput cross-linking immunoprecipitation (CLIP) assays as an example evolved to high throughput cross-linking immunoprecipitation (HITS-CLIP) and was used to map genome wide NOVA interactions described previously [80].

## 5. Conclusions and Future Directions in Therapeutic Interventions for Splicing Disorders

Ultimately, the larger goal in studying the mechanisms of splicing disruption is to enable further research in therapies that reverse splicing defects. Of the three classes of splicing disorders, mutations that disrupt splicing in *cis* may be most amenable to therapy as its effects are limited to a single gene. Oligonucleotides and other RNA binding compounds have been used to rescue aberrant splice site choices *in vivo*. The precise strategy for correcting a *cis*-mutation depends on the type of aberrant splicing that arises. Many aberrant splicing events are caused by the unwanted binding of a spliceosome component to a pre-mRNA element. For example, a silent variation in SMN2 was hypothesized to create a binding site of the repressor hnRNPA1 which reduced the inclusion of exon 7 of SMN2 and caused spinal muscular atrophy [81,82]. The binding of modified oligonucleotides to nearby hnRNPA1 binding sites rescued splicing [82]. In a similar manner, cryptic splice sites can also be blocked by complementary oligonucleotides restoring usage of the appropriate splice site [83]. Oligonucleotides delivered into the cell can be modified, usually in the sugar or backbone, to increase nuclease resistance, specificity and to improve delivery to the target. Common modifications include morpholino oligomers, 2'-methoxyethoxy, 2'-O-methyl phosphorothioate and locked nucleic acid (LNA) [84]. Variations of oligonucleotide therapy have moved beyond simple steric hindrance of binding. Bi-functional oligonucleotides that combine a targeting sequence with a splicing enhancer have been shown to rescue the defective splicing of SMN2 *in vivo* [85]. Oligonucleotides do not necessarily have to target the affected exon.

Duchenne Muscular dystrophy is caused by Dystrophin gene mutations many of which induce frameshifting exon skipping events. Pharmaceutical oligonucleotides (named eteplirsen and drisapersen) were designed to restore the open reading frame by skipping additional exons. The resulting message contains internal deletions, but encodes a more functional dystrophin protein. During clinical trials the drugs were shown to improve some features of the disease (but not mobility) [86].

Small molecule therapy has been utilized as an alternate strategies for correcting splicing defects. Numerous FDA approved compounds bind bacterial ribosomal RNA (e.g., aminoglycosides). Screening compounds for their ability to increase exon inclusion in the SMN2 transcript yielded a tetracycline-like compound, PTK-SMA1 [87]. Not all compounds function by directly binding RNA. There are numerous examples of compounds that change splice isoform ratios by altering the expression of chromatin modifying factors (well reviewed in [86]). While certain aberrant splicing events may be altered by these types of changes, it is likely that many other factors will be affected.

Finally, strategies are also being developed to counter the gain of function toxic RNAs. Here a repeat expansion titrates a splicing factor from the cell, potentially affecting numerous splicing events. For example the CUG repeat expansions associated with Myotonic Dystrophy type 1 (DM1) are being targeted by antisense oligonucleotides that function through a variety of mechanisms [88,89,90,91,92,93,94]. Other approaches include designed compounds and endonucleases that recognize (CUG) repeats [95,96,97,98]. However, significant challenges associated with drug delivery remain and how effective all of these approaches will be in patients is still a major unanswered question.

With the growing awareness of RNA processing in disease, new efforts to diagnose, characterize and treat splicing defects are underway. As the cost of sequencing decreases, techniques such as RNA-seq are enjoying more widespread use. These approaches will undoubtedly reveal a significant role for aberrant splicing in human disease. It is difficult to predict what role oligonucleotides will play in future therapies. There are numerous challenges that deter large scale development of oligonucleotides as drugs (e.g., delivery issues, the small number of patients afflicted with a particular allele). However oligonucleotide therapies offer some key advantages that may speed their development. Unlike small molecule targeting, the principle of oligonucleotide specificity (nucleotide base pairing) is well understood. Oligonucleotide therapies also appear to be inherently more conservative and will likely not be hampered by the safety issues that halted previous attempts at gene therapy.
